# Differential Effects of Soil Moisture and Air Temperature on Vegetation Dynamics in Northwest China’s Warming and Wetting Region: An LSTM Modeling Approach

**DOI:** 10.3390/plants15101542

**Published:** 2026-05-19

**Authors:** Yajun Si, Junpo Yu, Geng Li, Jesus Carrera, Jiming Jin, Haihua Bai

**Affiliations:** 1 College of Water Resources and Architectural Engineering, Northwest A&F University, Yangling 712100, China; yajunsi@nwafu.edu.cn; 2 College of Resources and Environment, Yangtze University, Wuhan 430100, China; junpo_yu@163.com (J.Y.); gengli.stu@yangtzeu.edu.cn (G.L.); 3 Institute of Environmental Assessment and Water Research (IDAEA), Consejo Superior de Investigaciones Científicas (CSIC), Jordi Girona 18, 08034 Barcelona, Spain; jesus.carrera.ramirez@gmail.com; 4 Institute of Grassland Research, Chinese Academy of Agricultural Sciences, Hohhot 010010, China; baihaihua@caas.cn; 5 Key Laboratory of Grassland and Agricultural Ecological Remote Sensing, Ministry of Agriculture and Rural Affairs, Hohhot 010010, China

**Keywords:** LAI, warming–wetting trend, LSTM, soil moisture, northwest China

## Abstract

Under the pronounced warming–wetting trend in Northwest China, understanding vegetation responses to the redistribution of hydrothermal resources is essential for interpreting regional ecohydrological processes. Here, we developed a bivariate Long Short-Term Memory (LSTM) model to simulate leaf area index (LAI) dynamics for four representative vegetation types (cold temperate forest, shrubland, grassland, and cropland), using air temperature and soil moisture as predictors. The model reproduces seasonal vegetation phenology well across vegetation types (R^2^ > 0.9), indicating that LSTM effectively captures the cumulative and lagged effects of hydrothermal drivers. However, its performance diverges at the interannual scale. Interannual variability in grasslands in water-limited environments is reasonably represented (R^2^ = 0.31), consistent with their sensitivity to short-term hydroclimatic variability under warming–wetting conditions. In contrast, the model fails to reproduce the observed long-term greening trend in forests when driven solely by hydrothermal variables. This contrast suggests distinct underlying mechanisms across ecosystem types. Grassland dynamics are closely linked to high-frequency hydroclimatic variability, whereas forest growth appears to be governed by slower processes and low-frequency drivers, including CO_2_ fertilization, nitrogen deposition, and ecological inertia. As a result, hydrothermal variables alone are insufficient to explain long-term forest dynamics. Overall, these findings highlight a transition from water-limited to energy- and process-limited controls across vegetation types and underscore the limitations of purely climate-driven models. Integrating biogeochemical processes or process-based constraints into machine learning frameworks may therefore be necessary to improve predictions of long-term vegetation change under climate change.

## 1. Introduction

Vegetation acts as a critical link in the Soil–Plant–Atmosphere Continuum (SPAC), playing an irreplaceable role in regulating the regional hydrological cycle, surface energy balance, and carbon budget [1,2,3]. Through processes such as evapotranspiration, canopy shading, and carbon assimilation, vegetation influences land–atmosphere interactions and serves as a vital feedback loop between terrestrial ecosystems and the climate system [4,5,6]. The leaf area index (LAI), a core biophysical parameter characterizing canopy structure and growth vitality, is widely used to describe vegetation productivity, evapotranspiration, and energy exchange. Its spatiotemporal evolution effectively reflects the response mechanisms of terrestrial ecosystems to climate change and hydrological processes [7,8,9].

This is particularly relevant in the ecologically fragile arid and semiarid regions of Northwest China, which have experienced a significant “warming–wetting” trend in recent decades [10,11,12]. Observational studies have reported a significant increase in air temperature, with warming rates of approximately 0.10–0.15 °C yr^−1^, accompanied by a concurrent increase in precipitation and soil moisture, typically on the order of 0.2–0.5 mm yr^−1^ [10,11,12]. Rising air temperatures provide abundant thermal resources, while increased precipitation improves critical moisture conditions. This combination of hydrothermal changes is considered a key driver of regional vegetation “greening” [9,13,14]. Satellite observations indicate a widespread greening trend in global arid and semiarid regions over the past few decades, with Northern China being particularly prominent [9,15,16]. In this context, structural changes in vegetation not only alter the regional carbon cycle but also affect water resource allocation and land surface hydrological processes through evapotranspiration [6,17,18]. Therefore, quantifying the driving mechanisms of hydrothermal factors on LAI variations under climate transition is of great significance for understanding ecohydrological processes in arid regions [19,20].

Vegetation growth is fundamentally controlled by the dynamic balance between energy and water. In arid and semiarid regions, soil moisture (SM), as the direct water source for roots, is a critical hydrological variable limiting vegetation productivity [2,21]. Meanwhile, near-surface air temperature at 2 m (Tair) directly affects photosynthetic enzyme activity, phenological processes, and the length of the growing season, serving as a vital thermal condition for growth [22,23]. Numerous studies have shown that the impact of hydrothermal conditions on vegetation growth is characterized by significant nonlinearity, spatial heterogeneity, and lagged responses [24,25,26]. For instance, in water-limited regions, soil moisture typically dominates productivity, whereas temperature often plays a more significant role in humid regions [27,28]. Furthermore, vegetation responses to climate drivers vary significantly by ecosystem type; forests, grasslands, and shrublands exhibit distinct sensitivities to hydrothermal changes [27,28,29].

Traditionally, academia has relied on physically based land surface models, such as the Community Land Model (CLM), to simulate land–atmosphere interactions and vegetation dynamics [30,31]. While these models describe photosynthesis, evapotranspiration, and carbon cycling through parameterized schemes and have played a crucial role in global climate research [32], they face challenges. Due to the complexity and nonlinearity of terrestrial ecosystem processes, traditional physical models still harbor significant uncertainties in parameterization and process representation [33]. Additionally, high-resolution simulations often incur massive computational costs, limiting their application in long time series or regional-scale studies [34].

In recent years, the advent of big data and artificial intelligence has provided new avenues for studying complex ecohydrological processes [33]. Among these, the Long Short-Term Memory (LSTM) network, a deep learning time series model, effectively captures long-term dependencies via gating structures [35]. Compared to traditional Recurrent Neural Networks (RNNs), LSTM mitigates the vanishing gradient problem and has demonstrated superior performance in climate prediction, hydrological modeling, and ecosystem dynamics [36,37]. Recent studies indicate that LSTM achieves high accuracy in simulating vegetation indices, the LAI, and soil moisture, revealing complex nonlinear relationships between climate factors and vegetation dynamics [38,39,40]. This end-to-end data-driven approach allows for the direct learning of mapping relationships from observational data, providing a novel tool for understanding vegetation dynamics under climate change [33].

Building on this, the present study focuses on the “warming–wetting” region of Northwest China, a typical climate-sensitive zone. We selected SM and Tair as key hydrothermal drivers to construct a bivariate LSTM model for simulating long-term LAI sequences. This study aims to address the following key aspects: (1) to reveal the spatiotemporal evolution of the LAI across different vegetation types under the warming–wetting trend; (2) to evaluate the applicability and advantages of the LSTM model in resolving hydrothermal coupling mechanisms; and (3) to explore the sensitivity differences in various vegetation types (e.g., forests, grasslands, and croplands) to changes in temperature and soil moisture. The findings will help deepen the understanding of ecohydrological response mechanisms in arid regions and provide a scientific basis for regional ecological restoration and water resource management.

## 2. Materials and Methods

### 2.1. Study Area

**Figure 1:**
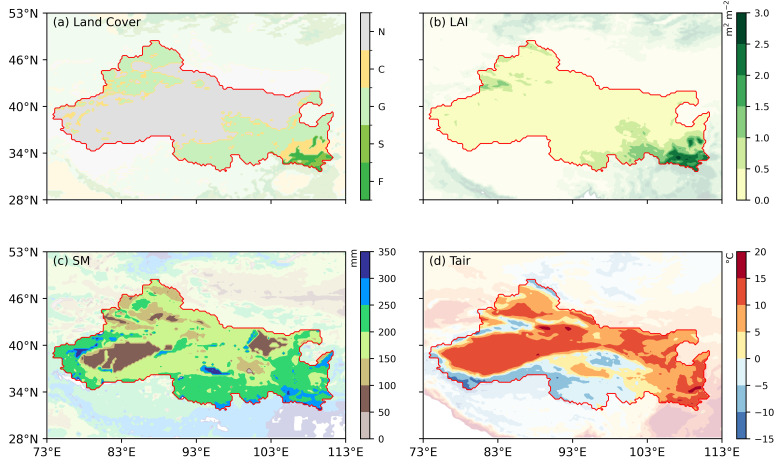
Spatial distribution of environmental variables in Northwest China during 2000–2021. (**a**) Land cover types, including forest (F), shrubland (S), grassland (G), cropland (C) and non-study area (N); (**b**) mean LAI (m^2^ m^–2^); (**c**) mean SM (mm); (**d**) mean Tair (°C). The study area is outlined in red. All variables are averaged over the study period.

Northwest China is located in the inland region of Eurasia (28–53° N, 73–113° E) and represents one of the driest areas at similar latitudes worldwide [41]. Owing to its continental location and the blocking effects of surrounding mountain ranges, the region receives very limited precipitation [42]. Annual mean precipitation is generally below 400 mm, and in some desert areas it is even less than 50 mm [43]. Together with strong evaporative demand, these conditions result in a typical arid to semiarid climate regime [44,45]. Under this climatic background, land cover in Northwest China exhibits pronounced spatial heterogeneity (Figure 1a). The central and western parts are dominated by extensive desert landscapes associated with the Tarim Basin and the Gobi Desert (light gray). With increasing moisture availability toward the east and south, large areas of grasslands (light green) gradually appear in the central-eastern and northern parts of the region. Croplands (yellow) are mainly distributed in the eastern and southeastern areas where water resources are relatively abundant and human activities are intensive, while small patches of shrubland (green) and temperate forests (dark green) occur in relatively humid high-elevation zones, such as the Tianshan Mountains in the west and the eastern margins of the study area. As a climate-sensitive region and an important ecological barrier of China, Northwest China has experienced a pronounced warming and wetting trend over recent decades [46]. Understanding the evolution of this climatic transition and its impacts on vegetation dynamics has therefore become a key issue in regional ecohydrological research [47].

### 2.2. Data Sources and Preprocessing

This study constructed a long-term pixel-level dataset based on multi-source remote sensing products and land surface assimilation data covering the period from 2000 to 2021. The dataset includes both meteorological forcing variables and vegetation characteristics. Vegetation dynamics were represented using the reprocessed MODIS Version 6.1 LAI dataset developed by Lin et al. [48]. This dataset has a temporal resolution of 8 days and a spatial resolution of 0.25°. Through an improved quality control system and time series reconstruction algorithm, the dataset effectively reduces missing values and abnormal fluctuations caused by cloud or aerosol contamination. This improvement significantly enhances the reliability of weak vegetation signals in arid and semiarid regions and has been widely validated in land surface and climate modeling studies [49,50]. Meteorological forcing variables were obtained from the Global Land Data Assimilation System (GLDAS–2.1) Noah model daily product with a spatial resolution of 0.25° [51,52]. Two key variables were selected to represent hydrothermal conditions: Tair and SM within the 0–100 cm soil layer. The 0–100 cm soil moisture was calculated by summing the first three soil layers provided by the Noah model (0–10 cm, 10–40 cm, and 40–100 cm). GLDAS datasets have been widely used in global and regional hydrological studies and have demonstrated good reliability and applicability [53,54,55].

At the same time, the spatial heterogeneity of the underlying surface was characterized using the MODIS land cover product (MCD12Q1) based on the IGBP classification scheme [56]. The original land cover classes were reclassified into five core ecosystem types: forest, shrubland, grassland, cropland, and non-study area. Specifically, deciduous broadleaf forests and mixed forests were grouped as forest; closed shrubland and open shrubland were combined as shrubland; savannas, woody savannas and grasslands were merged as grassland; and croplands and cropland/natural vegetation mosaics were classified as cropland. Other land cover types, including permanent wetlands, urban and built-up areas, snow and ice, barren or sparsely vegetated areas, and water, were classified as non-study areas.

To ensure strict alignment among heterogeneous datasets before being used as inputs to the deep learning framework, standardized preprocessing procedures were applied. In terms of spatial consistency, both the LAI dataset and meteorological forcing variables already have a spatial resolution of 0.25°. Therefore, only the higher resolution MODIS land cover data were upscaled to 0.25° using bilinear interpolation. Pixels with maximum LAI values below 0.01 during the entire study period were identified as non-vegetated barren land and removed from the dataset. Non-vegetated pixels (LAI < 0.01) were excluded from subsequent analyses using a vegetation mask, ensuring that the model focuses on vegetated regions and avoids biases introduced by the large fraction of desert and sparsely vegetated areas in Northwest China. This procedure effectively prevents the model from learning spurious growth signals caused by interpolation artifacts [57]. For temporal alignment, a sliding window approach was applied to reorganize the daily SM and Tair time series into feature tensors. In this way, each 8-day LAI observation was associated with the preceding high-frequency hydrothermal variability during the vegetation growth period. Finally, all variables were normalized to the range [0, 1] using min–max normalization in order to accelerate gradient convergence of the LSTM network and eliminate biases caused by differences in physical units [33].

### 2.3. LSTM-Based Vegetation Dynamic Modeling Framework

#### 2.3.1. Sample Construction and Network Architecture

Vegetation response to climate variability is not instantaneous but deeply constrained by the memory and cumulative effects of antecedent hydrothermal conditions [27]. LSTM networks, with their internal cell and gating mechanisms, effectively resolve the vanishing gradient problem inherent in traditional RNNs, thereby capturing nonlinear long-term dependencies in multivariate time series [35]. This method has been successfully applied to simulate global-scale ecosystem memory effects and complex hydrological processes [36,58]. Based on prior sensitivity tests and the existing literature [59], a lookback time window of 56 days was established. Each input sample comprises daily SM and Tair for the 56 consecutive days preceding the target date, designed to fully encompass the lagged impact of meteorological forcing on the LAI.

The bivariate LSTM modeling framework developed in this study consists of a single LSTM layer followed by a fully connected decoding layer. The hidden layer contains 256 neurons. To reduce the risk of overfitting caused by high dimensional parameters, dropout regularization with a dropout rate of 0.2 was applied between recurrent units [60]. A fully connected layer with 64 neurons was added after the LSTM layer to perform nonlinear mapping of high-dimensional temporal features, and the final output corresponds to the predicted LAI value at the target time step.

#### 2.3.2. Training Strategy and Simulation Experiments

To systematically decouple the driving mechanisms of different hydrothermal factor combinations on vegetation dynamics and evaluate LSTM input sensitivity, three independent control experiment schemes were designed: (1) Bivariate Model (bivariate)—Coupling SM and Tair as joint inputs to capture hydrothermal synergistic effects; (2) Univariate Moisture Model (univariate–SM)—Using SM as the sole input; (3) Univariate Temperature Model (univariate–Tair)—Using Tair as the sole input.

To implement these experiments, considering that vegetation responses across large regions exhibit both spatial heterogeneity and general response patterns, we adopted a transfer learning strategy referred to as global pretraining and local fine-tuning. At the spatial level, samples from all valid grid cells in the study area were first aggregated to construct a large pretraining dataset, enabling the model to capture general cumulative responses to hydrothermal forcing across different vegetation types [33,61]. Subsequently, the pretrained model was fine-tuned at each individual grid cell using local data. This design preserves local ecological specificity while substantially reducing the computational cost associated with independent training at each grid cell and improving spatiotemporal generalization. This approach allows the model to balance regional generality and local specificity in representing vegetation and climate interactions.

In the temporal dimension, to rigorously prevent data leakage [62], the full time series was split in chronological order. The first 80% of the data (2000–2017) was used for training, with a validation subset selected from this period, while the remaining 20% (2018–2021) was reserved as an independent test set for model evaluation. Model parameters were optimized using the Adam optimizer [63] as implemented in PyTorch version 2.1 (Meta AI, Menlo Park, CA, USA), with mean squared error (MSE) as the loss function. The initial learning rate was set to 0.001, with an adaptive decay strategy based on validation performance. In addition, an L2 regularization term of 1 × 10^−4^ was introduced to reduce model complexity. An early stopping mechanism was implemented based on validation metrics, with training automatically terminated within a maximum of 200 epochs to retain the optimal model parameters and avoid overfitting [64]. Model selection was primarily based on validation performance during the training phase, while the independent test set was used exclusively for evaluating model generalization. Given that the test period (2018–2021) is relatively short, it is not sufficient for robust climatological analysis of long-term trends and variability. After determining the optimal model configuration, the model was retrained using the full dataset (2000–2021) to generate continuous simulations across the study period. This step allows full utilization of available data for analyzing seasonal cycles, interannual variability, and long-term vegetation dynamics, while maintaining a clear separation between model evaluation and final simulation.

### 2.4. Statistical Evaluation and Long-Term Trend Analysis

To quantitatively evaluate the model’s performance in capturing both seasonal vegetation dynamics and interannual variability, two statistical metrics were used: the coefficient of determination (R^2^) and the root mean square error (RMSE). The coefficient of determination represents the proportion of variance in the observed LAI explained by the model [65], while the RMSE quantifies the average magnitude of prediction errors [66].

Let *S_i_* denote the simulated LAI value, *O_i_* the observed MODIS LAI value, and *N* the total number of observations. $\bar{S}$ and $\bar{O}$ denote the mean values of the simulated and observed LAI, respectively. The coefficient of determination is calculated as follows:

$$R^{2}={\left(\frac{\sum_{i=1}^{N}\left(S_{i}-\bar{S})(O_{i}-\bar{O}\right)}{\sqrt{\sum_{i=1}^{N}{\left(S_{i}-\bar{S}\right)}^{2}}\sqrt{\sum_{i=1}^{N}{\left(O_{i}-\bar{O}\right)}^{2}}}\right)}^{2}$$

R^2^ ranges from 0 to 1, although it may be negative in some cases. Values closer to 1 indicate a stronger agreement between simulated and observed values [67].

The RMSE is defined as follows:

$$RMSE=\sqrt{\frac{1}{N}\sum_{i=1}^{N}{\left(S_{i}-O_{i}\right)}^{2}}$$

Lower RMSE values indicate better agreement between simulated and observed values [68].

In addition, to investigate long-term vegetation dynamics under the warming–wetting climate background and to evaluate the capability of the LSTM model to capture low-frequency variability, trend analysis was conducted for both the observed and simulated LAI time series. Sen’s slope estimator was used to quantify the magnitude of monotonic trends [69], while the Mann–Kendall test was applied to assess their statistical significance [70]. Spatial trend patterns were derived by applying these methods to each grid cell. A positive Sen’s slope indicates a greening trend, whereas a negative slope indicates vegetation degradation [69,70]. Statistical significance was evaluated using the Mann–Kendall test at the *p* < 0.05 level [70]. By comparing the spatial patterns and magnitudes of trend slopes derived from observations and model simulations, this analysis allows us to assess whether the LSTM model can accurately reproduce vegetation trajectories driven by long-term climate change after removing seasonal variability. It also helps diagnose potential biases associated with missing drivers in the modeling framework, such as CO_2_ fertilization effects and nitrogen deposition [9,71].

## 3. Results

### 3.1. Spatiotemporal Characteristics of Environmental Variables

Figure 1b–d show the spatial distribution of the long-term mean LAI, SM, and Tair across Northwest China. The LAI exhibits pronounced spatial heterogeneity. The central and western regions are dominated by desert landscapes with LAI values close to zero, whereas grasslands and scattered croplands are mainly distributed along desert margins. Higher LAI values (>3 m^2^ m^−2^) are observed in the southeastern region, where forests and shrublands are concentrated, reflecting strong contrasts in vegetation cover across arid and semiarid environments. Soil moisture shows a clear increasing gradient from west to east and from inland to marginal regions. High values (200–300 mm yr^−1^) are mainly located in the eastern and southeastern parts of the study area, exhibiting strong spatial correspondence with regions of high LAI. In contrast, near-surface air temperature displays a different spatial pattern, primarily controlled by latitude and topography. The region generally transitions from cold conditions in the high-elevation and high-latitude areas of the west and north (annual mean temperature < 0 °C) to warmer conditions in the low-elevation and low-latitude areas of the east and south (annual mean temperature > 15 °C). For example, mountainous regions in the west and south are relatively cool, whereas basins and plains in the central and eastern regions are comparatively warm. Overall, the spatial patterns of the LAI, SM, and Tair differ substantially, highlighting strong spatial contrasts in vegetation, moisture availability, and thermal conditions across Northwest China. These results confirm that the warming–wetting region is characterized by complex and sensitive ecological conditions, with pronounced spatial gradients in key environmental variables.

**Figure 2:**
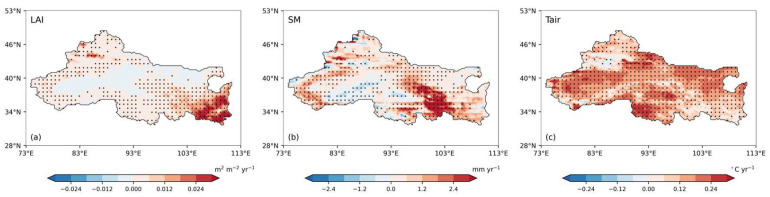
Spatial trends in key ecohydrological variables in Northwest China under a warming–wetting climate during 2000–2021. (**a**) LAI trend (m^2^ m^−2^ yr^−1^); (**b**) SM trend (mm yr^−1^); (**c**) Tair trend (°C yr^−1^). Color shading represents the magnitude and direction of the trends, and black dots indicate areas with statistically significant trends (*p* < 0.05).

Figure 2 presents the spatial trends of the LAI, SM, and Tair during 2000–2021. The LAI shows a significant overall increasing trend (mean 0.005 m^2^ m^−2^ yr^−1^, range from −0.033 to 0.054), with 59.1% of the area exhibiting significant trends (*p* < 0.05). Increasing trends are mainly observed in vegetated regions of the east and southeast, as well as in western oasis areas. Soil moisture exhibits strong spatial heterogeneity (mean 0.375 mm yr^−1^, range from −13.627 to 7.258 mm yr^−1^), with 46.3% of the area showing significant trends. Wetting dominates in the eastern and southern regions, whereas localized drying occurs in parts of the west and north, indicating heterogeneous hydrological responses to climate change. Tair shows a widespread warming trend (mean 0.113 °C yr^−1^, range from −0.237 to 0.538), with 64.0% of the area experiencing significant increases, further confirming the warming–wetting characteristics of the region. The combined changes in these variables suggest that increasing temperature and changing soil moisture conditions jointly drive vegetation recovery and hydrological processes. Regions in the east and southeast exhibit concurrent increases in the LAI, SM, and Tair, reflecting typical warming–wetting conditions, whereas parts of the northwest show warming accompanied by drying, which may be related to enhanced evapotranspiration, reduced glacier meltwater, or groundwater extraction [44,72]. Areas without significant trends may represent transition zones or regions dominated by background variability [73].

**Figure 3:**
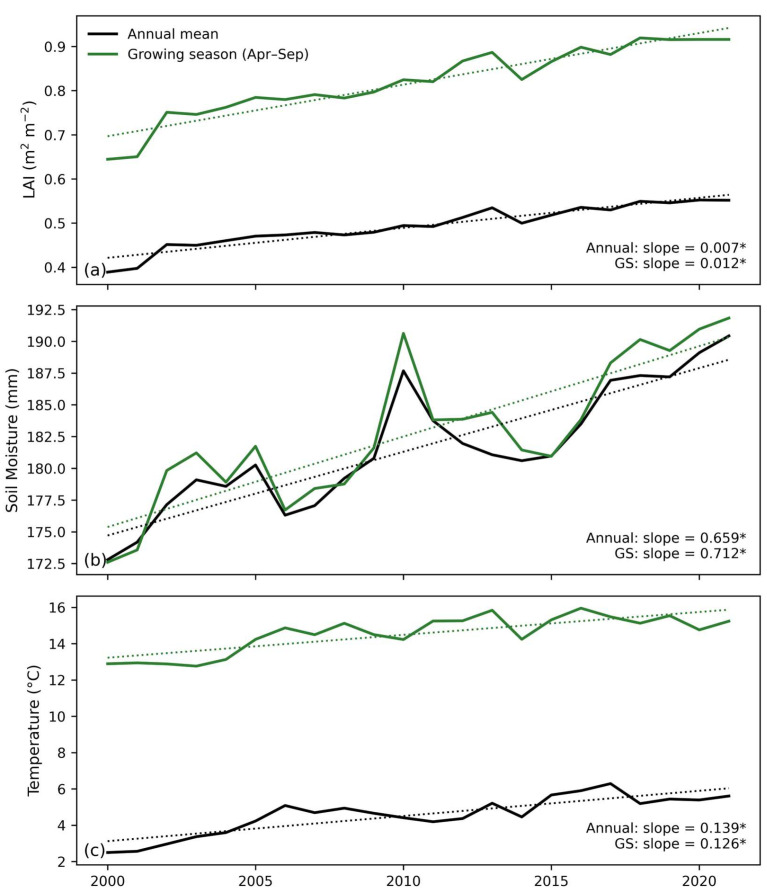
Interannual variations and trends in key ecohydrological variables in the northwestern humidification region of China during 2000–2021. (**a**) LAI (m^2^ m^−2^), (**b**) SM (mm), and (**c**) Tair (°C) shown as annual means (black lines) and growing season means (April–September, green lines). Solid lines represent yearly values and dashed lines show linear trends. Estimated slopes represent the rate of change per year, with asterisks (*) indicating statistical significance at *p* < 0.05.

Figure 3 shows the interannual variations in the LAI, soil moisture, and temperature during 2000–2021, including both annual means and growing season (April–September) averages. Both the annual and growing season LAIs exhibit significant increasing trends. The annual mean LAI increases at a rate of 0.007 m^2^ m^−2^ yr^−1^, while the growing season LAI shows a stronger increase of 0.012 m^2^ m^−2^ yr^−1^, both of which are highly significant (*p* < 0.05). These results provide strong evidence for vegetation greening in Northwest China, particularly during the peak growing season. Soil moisture also shows significant increasing trends, with annual mean and growing season slopes of 0.659 mm yr^−1^ and 0.712 mm yr^−1^, respectively, both highly significant (*p* < 0.05). This indicates an overall wetting trend, which provides favorable moisture conditions for vegetation growth. Although a pronounced peak in soil moisture occurred around 2009–2010, the long-term increasing trend remains evident. Air temperature exhibits a consistent upward trend, with annual mean and growing season increases of 0.139 °C yr^−1^ and 0.126 °C yr^−1^, respectively, both statistically significant (*p* < 0.05). This confirms a sustained warming trend across the region. The increase in temperature enhances thermal accumulation for vegetation growth, but may also intensify evapotranspiration and pose challenges to regional water balance.

### 3.2. Performance of LSTM Models in Simulating LAI

To assess the performance of LSTM models under different driver combinations, three model configurations were developed: a bivariate model using both SM and Tair as inputs, a univariate soil moisture model (univariate–SM), and a univariate air temperature model (univariate–Tair). Model performance was evaluated against MODIS LAI observations.

**Figure 4:**
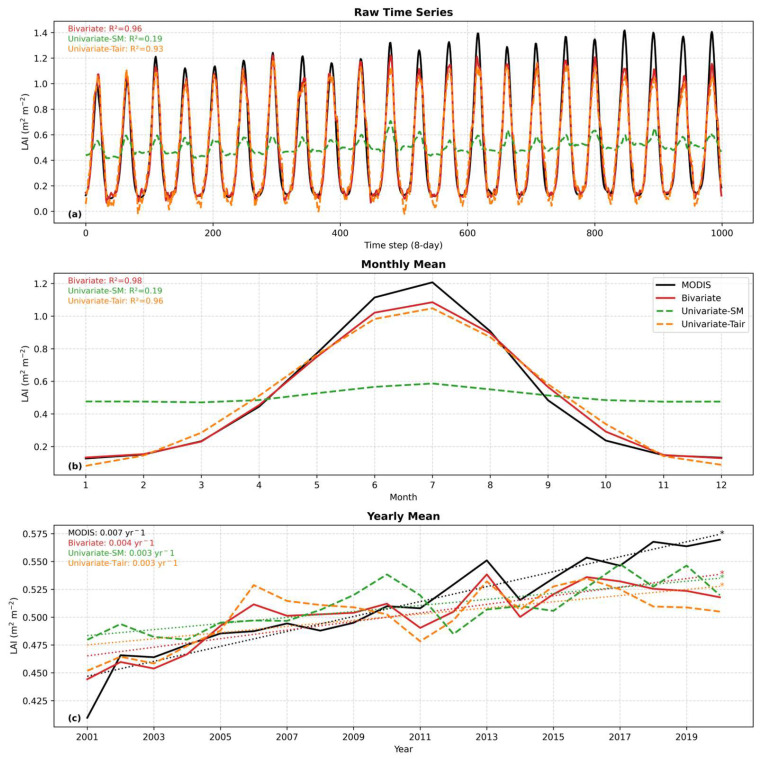
Comparison between simulated and observed LAI in Northwest China under a warming–wetting climate during 2000–2021. (**a**) Raw time series of LAI (8–day), (**b**) monthly mean LAI, and (**c**) yearly mean LAI. Three simulation schemes, including bivariate (SM + Tair, red), univariate–SM (green), and univariate–Tair (orange), are evaluated against MODIS LAI observations (black). Coefficients of determination (R^2^) are shown in panels (**a**,**b**). Linear trends in (**c**) represent the annual rate of change. Asterisks (*) indicate statistically significant relationships (*p* < 0.05).

Figure 4 compares the simulated and observed LAI time series under different input configurations. At both the original 8-day scale (Figure 4a) and the monthly scale (Figure 4b), the bivariate model shows strong agreement with the MODIS LAI, closely matching both the phase and amplitude of seasonal variations. The model effectively captures the rapid increase in the LAI during spring and summer, as well as the decline during autumn and winter, with R^2^ values of 0.96 and 0.98, respectively. In terms of a quantitative trend, the fitted slope for the bivariate model at the yearly scale approaches that of MODIS (0.004 vs. 0.007), further indicating its skill in capturing the secular greening trend (Figure 4c). In contrast, the univariate models show clear limitations. The univariate–Tair model reproduces the general seasonal cycle but exhibits noticeable deviations at peak values (R^2^ = 0.93 for Figure 4a and 0.96 for Figure 4b). The fitted trend slope (0.003 yr^−1^) remains lower than that of MODIS or the bivariate model, with an R^2^ of 0.44 at the interannual scale. The univariate–SM model produces a much smoother time series and fails to capture the seasonal dynamics of vegetation growth, yielding a low R^2^ of 0.19 at the 8-day and monthly scales. At the interannual scale, its fitted slope (0.003 yr^−1^) is minimal and R^2^ further declines to 0.45, indicating poor representation of observed interannual variability and long-term changes. Together, these metrics demonstrate that incorporating both SM and Tair not only substantially improves agreement with the observed LAI at multiple temporal scales—reflected by higher R^2^ values and more realistic trend slopes—but also enhances the model’s ability to track both short-term phenological dynamics and long-term greening trends.

**Figure 5:**
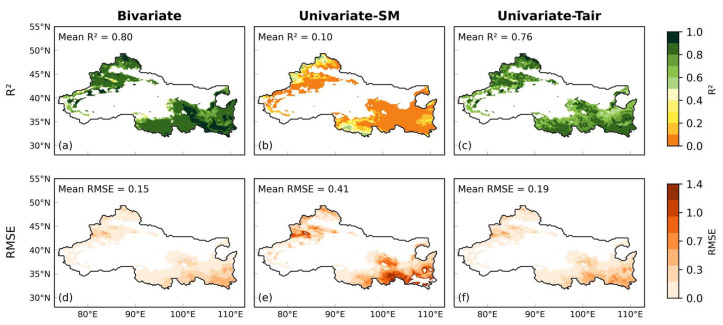
Spatial distribution of model performance across Northwest China. The upper row (**a**–**c**) shows the coefficient of determination (R^2^), and the lower row (**d**–**f**) shows the root mean square error (RMSE) for the (**a**,**d**) bivariate, (**b**,**e**) univariate–SM, and (**c**,**f**) univariate–Tair models. White areas indicate non-study regions. Color bars represent the corresponding metric values.

Figure 5 presents the spatial distribution of the R^2^ and RMSE for the three models, along with their corresponding regional mean values. The bivariate model (Figure 5a) achieves relatively high R^2^ values (mostly > 0.6) across most of the study area, particularly in the central and eastern vegetated regions, indicating strong agreement with observations. Its regional mean R^2^ reaches 0.80, substantially higher than that of the two univariate models. In contrast, the univariate–SM model (Figure 5b) shows generally lower R^2^ values (mostly 0.2–0.4) with a more scattered spatial pattern, and a markedly lower regional mean R^2^ of 0.10. The univariate–Tair model (Figure 5c) performs better than the univariate–SM model, with a regional mean R^2^ of 0.76, but still exhibits relatively low values in parts of the central and western regions, with higher values mainly concentrated in the east. The RMSE distributions (Figure 5d–f) further highlight these differences. The bivariate model shows relatively low RMSE values (mostly 0.0–0.3) across most regions, indicating higher simulation accuracy, with a regional mean RMSE of 0.15. In contrast, both univariate models exhibit higher RMSE values, particularly in sparsely vegetated areas, suggesting larger simulation errors. The univariate–SM model has the highest regional mean RMSE (0.41), while the univariate–Tair model shows moderate performance with a mean RMSE of 0.19, still higher than that of the bivariate model.

**Figure 6:**
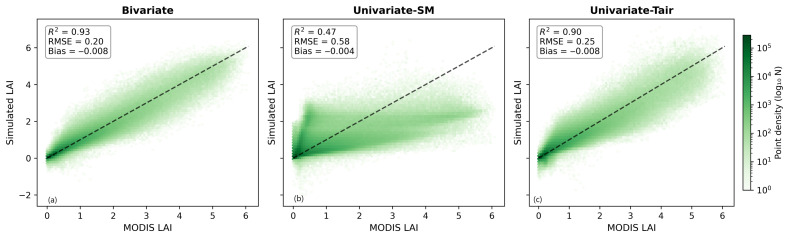
Hexagonal bin density plots comparing simulated LAI with MODIS LAI for three LSTM models: (**a**) bivariate, (**b**) univariate–SM, and (**c**) univariate–Tair. Color shading represents the logarithmic density of data points. The dashed line denotes the 1:1 reference line. Values of R^2^, RMSE, and bias are shown in each panel.

Furthermore, Figure 6 provides a quantitative assessment of overall model performance using density plots comparing the simulated LAI with the MODIS LAI. The bivariate model (Figure 6a) achieves the best performance, with an R^2^ of 0.93, an RMSE of 0.20, and a bias of −0.008. The data points are tightly clustered around the 1:1 line, indicating strong agreement between simulations and observations. In contrast, the performance of the univariate–SM model (Figure 6b) is substantially lower, with an R^2^ of 0.47, an RMSE of 0.58, and a bias of −0.004. The scatter distribution shows considerable dispersion at low LAI values and clear underestimation at higher LAI levels. The univariate–Tair model (Figure 6c) shows intermediate performance, with an R^2^ of 0.90, an RMSE of 0.25, and a bias of −0.008. Although the overall fit is relatively strong, deviations are evident in the low LAI range, particularly near zero. In addition, the simulated LAI does not fully span the observed range, especially at higher values, which is consistent with the reduced amplitude observed in the time series (Figure 4a).

### 3.3. LAI Variations Across Different Vegetation Types

**Figure 7:**
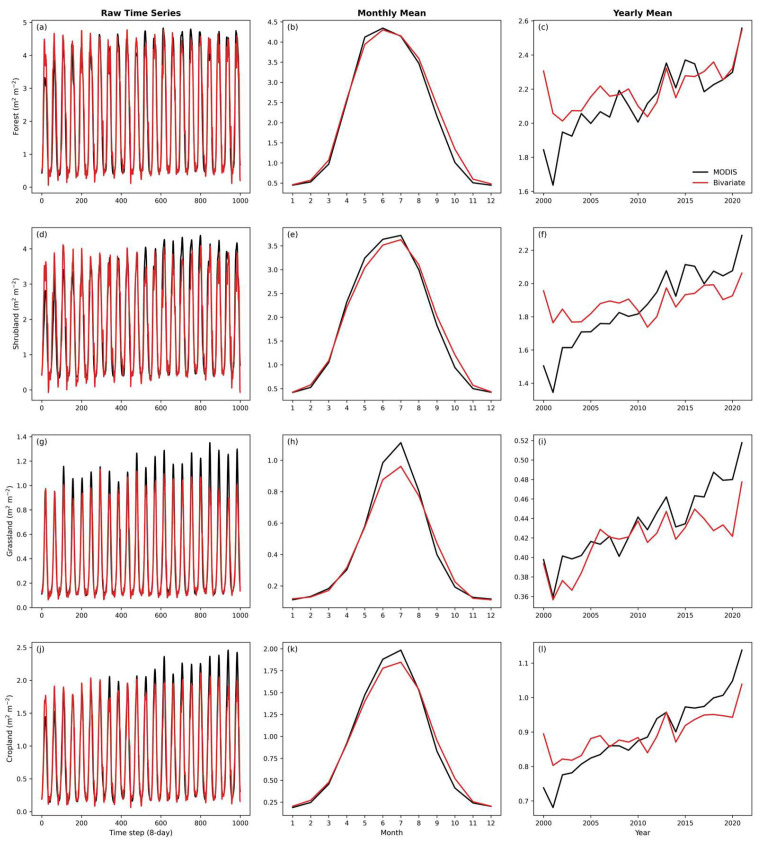
Comparison between MODIS-observed and bivariate model-simulated LAI for four vegetation types in Northwest China under a warming–wetting climate during 2000–2021. (**a**–**c**) Forest, (**d**–**f**) shrubland, (**g**–**i**) grassland, and (**j**–**l**) cropland. Panels show (**a**,**d**,**g**,**j**) 8-day LAI time series; (**b**,**e**,**h**,**k**) monthly mean seasonal cycles; and (**c**,**f**,**i**,**l**) annual mean trends. Black lines denote MODIS LAI observations, and red lines represent bivariate model simulations.

To examine vegetation-specific responses under the warming–wetting background in Northwest China, the performance of the bivariate model was evaluated across four representative vegetation types (forest, shrubland, grassland, and cropland), and their LAI dynamics were analyzed at multiple temporal scales. Figure 7 presents LAI variations at the original 8-day scale (first column of Figure 7), monthly scale (second column of Figure 7), and annual scale (third column of Figure 7). Overall, the bivariate model captures both seasonal variability and interannual trends of the LAI across all vegetation types. At the original time series scale, the simulated LAI (red line) closely matches the MODIS LAI (black line), reproducing the annual cycle of vegetation growth and senescence. At seasonal scales, the model shows a strong ability to capture phenological patterns, with the timing of peak growth closely aligned with observations. However, model performance varies among vegetation types. For forests and shrublands, the model accurately reproduces the peak LAI values during June and July. In contrast, for grasslands and croplands, although seasonal patterns are well represented, peak values are slightly underestimated. For example, the observed peak LAI for grasslands is approximately 1.15 m^2^ m^−2^, whereas the simulated value is about 0.95 m^2^ m^−2^. A similar underestimation is found in croplands (approximately 2.0 vs. 1.85 m^2^ m^−2^). This pattern indicates that additional factors beyond temperature and soil moisture may influence the peak LAI in low-stature vegetation, particularly during rapid growth periods.

**Figure 8:**
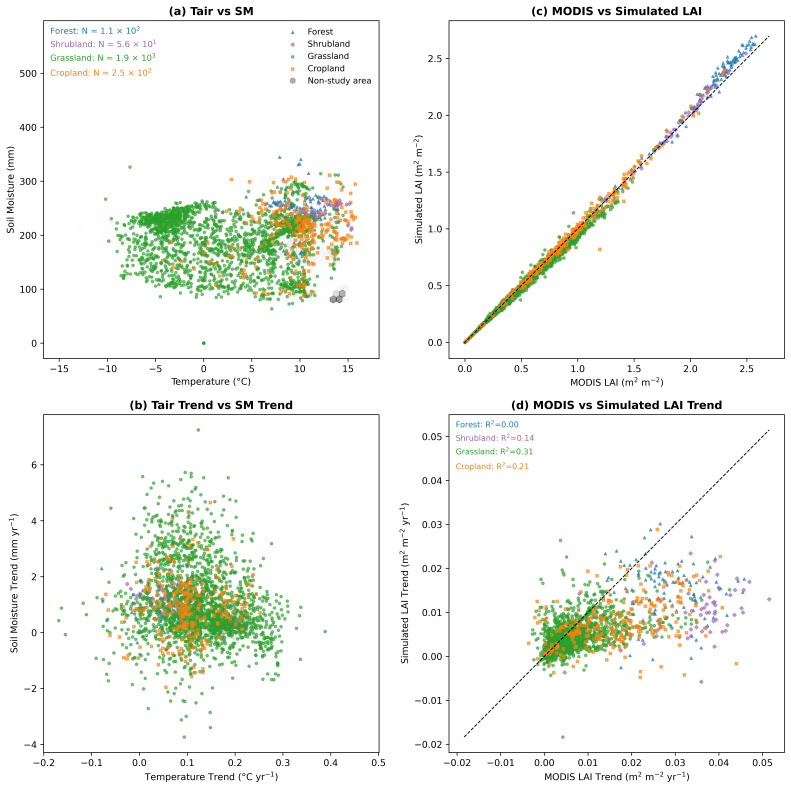
Relationships between air temperature, soil moisture, and leaf area index (LAI) across different vegetation types in Northwest China. (**a**) Relationship between air temperature and soil moisture. Sample size (N) for each vegetation type is shown. (**b**) Relationship between temperature trend and soil moisture trend. (**c**) Comparison between MODIS LAI and simulated LAI. (**d**) Comparison between MODIS LAI trend and simulated LAI trend; the coefficient of determination (R^2^) for each vegetation type is indicated. Different colors represent vegetation types, including forest, shrubland, grassland, and cropland. Non-study areas are shown as gray hexagonal bins, with the shade of gray representing background point density. The dashed lines in (**c**,**d**) indicate the 1:1 relationship.

At the interannual scale (Figure 7c), all vegetation types exhibit a clear greening trend since 2000, although their trajectories differ. Forests show the most pronounced and nearly linear increase, with annual mean LAI rising from about 1.6 m^2^ m^−2^ in the early 2000s to nearly 2.6 m^2^ m^−2^ by 2020. In contrast, grasslands and croplands display more fluctuating increases, with relatively high interannual variability (e.g., the grassland LAI ranges from 0.36 to 0.52 m^2^ m^−2^). The model captures these interannual variations to some extent, particularly for grasslands, where it reproduces the increase during 2015–2019. This behavior is consistent with the ecological niche distribution shown in Figure 8a, where grasslands are primarily located in relatively dry environments (SM < 200 mm) and are therefore more sensitive to variations in water availability. In contrast, forests are mainly distributed in relatively stable warm and moist environments (Tair > 10 °C, SM > 200 mm), where growth is less directly constrained by short-term moisture variability.

Figure 8 further examines the relationships between the LAI and climate drivers across vegetation types. Figure 8a shows the distribution of different vegetation types in temperature and soil moisture space, revealing clear clustering patterns. Grasslands are widely distributed across a broad range of relatively low soil moisture conditions (generally below ~250 mm) and span both low and moderate temperature regimes, indicating their prevalence in water-limited environments. In contrast, forests and woody vegetation are concentrated in a narrower range of warmer and wetter conditions, typically with higher temperatures (>10 °C) and soil moisture values above ~200 mm. Croplands occupy an intermediate range but tend to cluster toward relatively warm conditions, reflecting their dependence on both climatic conditions and human management. These distinct distributions highlight clear differences in hydroclimatic niches among vegetation types.

Figure 8b illustrates the relationship between temperature trends and soil moisture trends. The scatter plot shows considerable dispersion, indicating substantial spatial heterogeneity in hydroclimatic changes across the region. Most data points are concentrated in the positive temperature trend range, confirming widespread warming, while soil moisture trends exhibit both positive and negative values. Grasslands display the widest spread in soil moisture trends, including both wetting and drying signals, whereas forests and woody vegetation are more tightly clustered, suggesting relatively stable hydroclimatic conditions. This pattern indicates that different vegetation types are exposed to distinct combinations of temperature and moisture changes.

In terms of model performance, Figure 8c compares the vegetation-type-averaged simulated and observed LAI based on temporally averaged grid-cell values. The comparison is performed using all valid spatial pixels after averaging over the time dimension, showing a close agreement with most points distributed near the 1:1 line. However, when focusing on interannual trends (Figure 8d), model performance decreases notably. In particular, the model shows a limited ability to reproduce interannual variability for forests (R^2^ = 0.00) and shrubs (R^2^ = 0.14), whereas performance is relatively better for grasslands (R^2^ = 0.31) and croplands (R^2^ = 0.21). These results indicate that while the LSTM model captures mean LAI levels and seasonal dynamics effectively, its ability to represent interannual variability differs across vegetation types.

## 4. Discussion

This study confirms that, under the pronounced “warming–wetting” climate in Northwest China, the redistribution of hydrothermal resources strongly influences the spatiotemporal dynamics of the regional vegetation LAI. Different vegetation types respond in distinct ways, reflecting ecological niche differentiation and shifts between water-limited and energy-limited growth constraints.

Ecological niche analysis (Figure 8a) shows that grasslands are mainly distributed in regions with low to moderate SM, suggesting that they are generally exposed to water-limited conditions. Under such conditions, grassland ecosystems tend to exhibit strong sensitivity to variations in water availability, which plays an important role in regulating their LAI dynamics. During the warming–wetting period, grasslands exhibit a clear increasing trend in LAI. This pattern is unlikely to be explained by a single driver. Instead, it more likely reflects the combined effects of temperature and moisture, where warming enhances vegetation activity, while changes in water availability modulate the magnitude of this response. This interpretation is consistent with the interannual variations shown in Figure 7, where grasslands respond rapidly to wetting phases after 2010, and the model captures these dynamics to a certain extent (R^2^ = 0.31). These observations are consistent with previous reports of arid zone grasslands responding quickly to precipitation pulses [74,75,76]. In contrast, forests and shrubs occupy warmer and more humid areas. Rising air temperatures generally promote photosynthesis and longer growing seasons. However, as shown in Figure 8d, trends in Tair and soil moisture alone fail to explain long-term forest LAI dynamics (R^2^ = 0.00). Forest growth seems influenced by physiological regulation, ecological inertia, and low-frequency drivers such as CO_2_ fertilization and nitrogen deposition, which were not included in our bivariate model [9,77,78,79]. Therefore, hydrothermal variables alone are likely insufficient to explain forest LAI trends. Another important factor not included in this study is atmospheric CO_2_ concentration. Long-term increases in CO_2_ can enhance vegetation growth through fertilization effects and can also influence temperature via the greenhouse effect. The absence of this driver in the current framework likely contributes to the limited ability of the model to reproduce long-term vegetation trends, particularly in forest ecosystems.

Spatial evaluation (Figure 5) indicates that the bivariate model generally performs better than univariate models, showing higher R^2^ values and lower RMSE values in most vegetated regions. The univariate–SM model, in particular, struggled in sparsely vegetated areas. Regionally averaged statistics further highlight the advantage of combining soil moisture and air temperature for LAI simulation. Density-based assessments (Figure 6) reinforce this conclusion. Hexagonal bin plots show that the bivariate model reproduces the MODIS LAI along the 1:1 line, with high point density across both low and high LAI values. By contrast, univariate models exhibit more dispersed distributions, indicating weaker predictive skill. Seasonal dynamics (Figure 4a,b) show that the simulated LAI closely tracks observed intra-annual cycles across vegetation types, suggesting that the LSTM model effectively captures seasonal phenology. Temporally averaged spatial comparisons (Figure 8c) indicate that the model reproduces mean LAI patterns across grid cells with reasonable spatial consistency. However, interannual trends after removing seasonality (Figure 8d) differ among vegetation types: grasslands and croplands retain moderate explanatory power (R^2^ = 0.31 and 0.21, respectively), whereas forests fail to capture interannual anomalies (R^2^ = 0.00). In addition, the hydrological representation in this study is limited to soil moisture. While SM integrates precipitation, infiltration, and evapotranspiration processes and directly reflects plant water availability, other variables such as precipitation and plant available water (PAW) may provide complementary information. Including these variables could improve the representation of hydrological controls on vegetation dynamics and will be considered in future work. The relatively small magnitude of soil moisture trends compared to temperature and the LAI may also partly explain the stronger contribution of temperature in capturing LAI variability.

Although the analysis focuses on vegetated pixels within Northwest China, future studies could further refine regional definitions or extend the study area to include more densely vegetated regions. Taken together, the results point to differences in “greening” mechanisms among vegetation types under a warming–wetting climate. Grasslands respond quickly to hydrothermal changes, particularly increased water-limited conditions, allowing the LSTM model to capture interannual fluctuations. Forest growth, in contrast, appears driven by longer term processes and low-frequency factors such as CO_2_ fertilization, nitrogen deposition, and age-related ecological inertia. Limiting the model to hydrothermal inputs alone constrains trend attribution for forests [33,80,81]. Quantifying the relative contributions of temperature and soil moisture requires additional interpretability analysis, which is beyond the scope of this study. To address this limitation, we are currently developing an interpretable multivariate time series modeling framework based on the IMV-Tensor architecture, which integrates attention-based interpretability mechanisms and will enable explicit assessment of variable importance and temporal contributions in future work.

Even when relying solely on hydrothermal variables, the LSTM model achieves high accuracy in seasonal dynamics (R^2^ = 0.96) and effectively captures phenology, outperforming conventional physical models such as the CLM. For croplands, irrigation practices may play an important role in regulating vegetation growth and LAI dynamics. The absence of irrigation data in this study may introduce additional uncertainty in model performance for cropland areas. Nevertheless, purely data-driven approaches remain limited in reproducing long-term interannual trends governed by complex processes. Future work could explore hybrid models that integrate physical mechanisms with deep learning, such as physics-guided machine learning, incorporating key low-frequency drivers or process constraints. Such approaches may preserve high seasonal simulation accuracy while providing physically interpretable predictions for long-term trends.

## 5. Conclusions

This study applied a bivariate LSTM modeling framework to investigate vegetation dynamics and their climatic drivers under the warming–wetting background in Northwest China. The results indicate that although the region exhibits an overall greening trend, vegetation responses differ substantially among ecosystem types. The LSTM model reproduced the seasonal phenology of all vegetation types with high accuracy (R^2^ = 0.96), suggesting that hydrothermal conditions play a dominant role in regulating seasonal vegetation dynamics and that the model effectively captures the nonlinear and lagged responses between climate variables and vegetation growth.

At longer temporal scales, however, clear differences emerged among vegetation types. The model was able to reproduce the interannual variability of grasslands (R^2^ = 0.31), indicating that grassland dynamics are closely associated with short-term hydroclimatic variability and its interaction with temperature. In contrast, when driven solely by hydrothermal variables, the model failed to reproduce the long-term greening trend observed in forest ecosystems (R^2^ = 0.00). This contrast suggests that while improved hydrothermal conditions may contribute to the variability in shallow-rooted vegetation such as grasslands, they are insufficient to explain the sustained growth of forest ecosystems.

These findings imply that forest dynamics are likely influenced by slower ecological and biogeochemical processes that were not included in the model, such as CO_2_ fertilization, nitrogen deposition, and ecological conservation measures. While the LSTM framework provides an effective tool for capturing seasonal phenology and short-term vegetation variability, improving the understanding and prediction of long-term ecosystem dynamics will require integrating additional ecological processes into machine learning models. Incorporating key biogeochemical drivers and process-based constraints may therefore help improve both attribution and predictive performance in studies of vegetation responses to ongoing climate change.

## Data Availability

Data are available in a publicly accessible repository. The GLDAS dataset is openly available at https://disc.gsfc.nasa.gov/datasets?keywords=GLDAS&page=1 (accessed on 30 March 2025). The data presented in this study are openly available: The LAI dataset is openly available at http://globalchange.bnu.edu.cn/research/laiv061 (accessed on 30 March 2025). The Landcover dataset is openly available at https://www.earthdata.nasa.gov/data/catalog/lpcloud-mcd12q1-061 (accessed on 30 March 2026).

## References

[1] Bonan G.B. (2008). Forests and climate change: Forcings, feedbacks, and the climate benefits of forests. Science.

[2] Seneviratne S.I., Corti T., Davin E.L., Hirschi M., Jaeger E.B., Lehner I., Orlowsky B., Teuling A.J. (2010). Investigating soil moisture–climate interactions in a changing climate: A review. Earth-Sci. Rev..

[3] Piao S., Liu Q., Chen A., Janssens I.A., Fu Y., Dai J., Liu L., Lian X., Shen M., Zhu X. (2019). Plant phenology and global climate change: Current progresses and challenges. Glob. Chang. Biol..

[4] Beer C., Reichstein M., Tomelleri E., Ciais P., Jung M., Carvalhais N., Rödenbeck C., Arain M.A., Baldocchi D., Bonan G.B. (2010). Terrestrial gross carbon dioxide uptake: Global distribution and covariation with climate. Science.

[5] Jung M., Reichstein M., Ciais P., Seneviratne S.I., Sheffield J., Goulden M.L., Bonan G.B., Chen A.A., Davis K.J., Gleason J.D. (2010). Recent decline in the global land evapotranspiration trend due to limited moisture supply. Nature.

[6] Forzieri G., Alkama R., Miralles D.G., Cescatti A. (2017). Emerging signals of declining photosynthesis in water-limited environments. Nat. Commun..

[7] Myneni R.B., Hoffman S., Knyazikhin Y., Privette J.L., Glassy J., Tian Y., Wang Y., Song X., Zhang Y., Smith G.R. (2002). Global products of vegetation leaf area and fraction absorbed PAR from year one of MODIS data. Remote Sens. Environ..

[8] Running S.W., Nemani R.R., Heinsch F.A., Zhao M., Reeves M., Hashimoto H. (2004). A continuous satellite-derived measure of global terrestrial primary production. BioScience.

[9] Zhu Z., Piao S., Myneni R.B., Huang M., Zeng Z., Canadell J.G., Ciais P., Sitch S., Friedlingstein P., Arneth A. (2016). Greening of the Earth and its drivers. Nat. Clim. Chang..

[10] Chi H., Wu Y., Zheng H., Zhang B., Sun Z., Yan J., Ren Y., Guo L. (2023). Spatial patterns of climate change and associated climate hazards in Northwest China. Sci. Rep..

[11] Chen Y., Li Z., Fan Y., Wang H., Deng H. (2015). Progress and prospects of climate change impacts on hydrology in the arid region of northwest China. Environ. Res..

[12] Huang J., Li Y., Fu C., Chen F., Fu Q., Dai A., Shinoda M., Ma Z., Guo W., Li X. (2017). Dryland climate change: Recent progress and challenges. Rev. Geophys..

[13] Piao S., Wang X., Ciais P., Zhu B., Wang T., Liu J. (2011). Changes in satellite-derived vegetation growth trend in temperate and boreal Eurasia from 1982 to 2006. Glob. Chang. Biol..

[14] Meng N., Wang N.A., Cheng H., Liu X., Niu Z. (2023). Impacts of climate change and anthropogenic activities on the normalized difference vegetation index of desertified areas in northern China. J. Geogr. Sci..

[15] Donohue R.J., Roderick M.L., McVicar T.R., Farquhar G.D. (2013). Carbon dioxide fertilisation has increased leaf area in Earth’s arid environments. Geophys. Res. Lett..

[16] Piao S., Tan J., Chen A., Fu Y.H., Ciais P., Liu Q., Janssens I.A., Vicca S., Zeng Z., Jeong S.J. (2015). Leaf onset date predicted by models lacks long-term in situ validation data. Nat. Clim. Chang..

[17] Li W., Duveiller G., Wieneke S., Ciais P., Anderegg W.R.L., Ellsäßer I., Wigneron J.P., Yang H., Wang Y., Zhang Z. (2024). Regulation of the global carbon and water cycles through vegetation structural and physiological dynamics. Environ. Res. Lett..

[18] Fisher J.B., Melton F., Middleton E., Hain C., Anderson M., Allen R., McCabe M.F., Hook S., Baldocchi D., Townsend P.A. (2017). The future of evapotranspiration: Global requirements for ecosystem functioning, carbon and climate feedbacks, agricultural management, and water resources. Water Resour. Res..

[19] Nemani R.R., Keeling C.D., Hashimoto H., Jolly W.M., Piper S.C., Tucker C.J., Myneni R.B., Running S.W. (2003). Climate-driven increases in global terrestrial net primary production from 1982 to 1999. Science.

[20] Peng S., Piao S., Ciais P., Myneni R.B., Chen A., Chevallier F., Dolman A.J., Janssen I.A., Peñuelas J., Bousquet P. (2013). Asymmetric effects of daytime and night-time warming on Northern Hemisphere vegetation. Nature.

[21] Dorigo W.A., Wagner W., Albergel C., Albrecht F., Balsamo G., Brocca L., Chung D., Ertl M., Forkel M., Gruber A. (2017). ESA CCI soil moisture for improved Earth system understanding: State-of-the art and future directions. Remote Sens. Environ..

[22] Richardson A.D., Anderson R.S., Arain M.A., Barr A.G., Bohrer G., Chen G., Chen J.M., Ciais P., Davis K.J., Desai A.R. (2013). Terrestrial biosphere model performance in North America. Glob. Chang. Biol..

[23] Peñuelas J., Rutishauser T., Filella I. (2009). Phenology feedbacks on climate change. Science.

[24] Reichstein M., Bahn M., Ciais P., Frank D., Mahecha M.D., Seneviratne S.I., Zscheischler J., Beer C., Buchmann N., Frank D.C. (2013). Climate extremes and the carbon cycle. Nature.

[25] Zhang Y., Liu S., Hu X., Wang H., He X., Li C., Wang J. (2020). Evaluating spatial heterogeneity of land surface hydrothermal conditions in the Heihe River Basin. Chin. Geogr. Sci..

[26] Humphrey V., Zscheischler J., Ciais P., Gudmundsson L., Seneviratne S.I., Gudmundsson L. (2018). Sensitivity of atmospheric CO_2_ growth rate to observed changes in terrestrial water storage. Nature.

[27] Seddon A.W., Macias-Fauria M., Long P.R., Benz D., Willis K.J. (2016). Sensitivity of global terrestrial ecosystems to climate variability. Nature.

[28] Liu L., Gudmundsson L., Hauser M., Qin D., Li S., Seneviratne S.I. (2020). Soil moisture dominates dryness stress on ecosystem production globally. Nat. Commun..

[29] Zhang Y., Gentine P., Luo X., Lian X., Liu Y., Zhou S., Cai W., Joiner J., Peng S., Hua W. (2020). Increasing vegetation greenness in the drylands of China. Glob. Chang. Biol..

[30] Oleson K.W., Lawrence D.M., Bonan G.B., Flanner M.G., Kluzek E., Lawrence P.J., Levis S., Swenson S.C., Thornton P.E. (2010). Technical Description of Version 4.0 of the Community Land Model (CLM).

[31] Lawrence D.M., Fisher R.A., Koven C.D., Oleson K.W., Swenson S.C., Bonan G.B., Collier N., Ghimire B., van Kampenhout L., Kennedy D. (2019). The Community Land Model version 5 (CLM5): Description of new features, benchmarking, and impact of forcing uncertainty. J. Adv. Model. Earth Syst..

[32] Bonan G.B., Lawrence P.J., Oleson K.W., Levis S., Jung M., Reichstein M., Lawrence D.M., Swenson S.C. (2011). Improving canopy processes in the Community Land Model version 4 (CLM4) using global flux datasets. J. Geophys. Res. Biogeosci..

[33] Reichstein M., Camps-Valls G., Stevens B., Jung M., Denzler J., Carvalhais N., Prabhat (2019). Deep learning and process understanding for data-driven Earth system science. Nature.

[34] Best M.J., Pryor M., Clark D.B., Rooney G.G., Essery R.L., Ménard C.B., Edwards J.M., Hendry M.A., Porson A., Gedney N. (2011). The Joint UK Land Environment Simulator (JULES), model description—Part 1: Energy and water fluxes. Geosci. Model Dev..

[35] Hochreiter S., Schmidhuber J. (1997). Long short-term memory. Neural Comput..

[36] Kratzert F., Klotz D., Brenner C., Schulz K., Herrnegger M. (2018). Rainfall–runoff modelling using Long Short-Term Memory (LSTM) networks. Hydrol. Earth Syst. Sci..

[37] Sheng Z., Wu X., Liu Y., Zhang W., Chen Y. (2023). A survey on data-driven runoff forecasting models based on neural networks. IEEE Trans. Emerg. Top. Comput. Intell..

[38] Chattopadhyay A., Mustafa M., Hassanzadeh P., Bach E., Kashinath K. (2020). Analog forecasting of extreme-causing weather patterns using deep learning. J. Adv. Model. Earth Syst..

[39] Xiao G., Chen Y., Wang J., Li S., Liu S., Wang Y., Li Y., Li S., Lu X., Luo M. (2025). Progress and perspectives of crop yield forecasting with remote sensing: A review. IEEE Geosci. Remote Sens. Mag..

[40] Taheri M., Schreiner H.K., Mohammadian A., Shirkhani H., Payeur P., Imanian H., Cobo J.H. (2023). A review of machine learning approaches to soil temperature estimation. Sustainability.

[41] Zhao Y., Yu Z., He C., Ni Z., Wang X. (2019). The evolution of the arid climate in China. Earth-Sci. Rev..

[42] Yao J., Hu W., Chen X., Huo W., Zhao Y., Xu X., Li M. (2018). Precipitation and temperature trends in the arid region of Northwest China. Atmos. Res..

[43] Wang Y., Chen Y., Pan X., Wang H. (2021). The precipitation trend in the arid region of Northwest China. J. Hydrol..

[44] Huang J., Yu H., Guan X., Wang G., Guo R. (2016). Accelerated dryland expansion under climate change. Nat. Clim. Chang..

[45] Yong L., Zhu G., Wan Q., Xu Y., Zhang Z., Sun Z., Ma H., Sang L., Liu Y., Guo H. (2020). The soil water evaporation process from mountains based on the stable isotope composition in a Headwater Basin and Northwest China. Water.

[46] Shi Y., Shen Y., Hu R., Zhang G., Ding Y., Li D., Ding Y. (2002). Preliminary study on signal, impact and forecast of climatic shift from warm-dry to warm-humid in Northwest China. J. Glaciol. Geocryol..

[47] Li X., Chen Y., Wang J. (2021). Eco-hydrological processes and responses to climate change in Northwest China: An overview. Sci. Total Environ..

[48] Lin W., Yuan H., Dong W., Zhang S., Liu S., Wei N., Lu X., Wei Z., Hu Y., Dai Y. (2023). Reprocessed MODIS Version 6.1 Leaf Area Index Dataset and Its Evaluation for Land Surface and Climate Modeling. Remote Sens..

[49] Chen C., Park T., Wang X., Piao S., Xu B., Chaturvedi R.K., Fuchs R., Brovkin V., Ciais P., Fensholt R. (2019). China’s and India’s greening drives through land-use management. Nat. Sustain..

[50] Chen L., Ma Z., Zhao T. (2017). Modeling and analysis of the potential impacts on regional climate due to vegetation degradation over arid and semi-arid regions of China. Clim. Change.

[51] Beaudoing H.K., Rodell M. (2016). GLDAS Noah Land Surface Model L4 3 Hourly 0.25 x 0.25 Degree V2.1.

[52] Rodell M., Houser P.R., Jambor U., Gottschalck J., Mitchell K., Meng C.-J., Arsenault K., Cosgrove B., Radakovich J., Bosilovich M. (2004). The global land data assimilation system. Bull. Am. Meteorol. Soc..

[53] Wang L., Shi X., Zhang T., Wu H., Wang X. (2016). Evaluation of the GLDAS-2.1 datasets using ground-based observations in the Tibetan Plateau. J. Geophys. Res. Atmos..

[54] Awange J.L., Gebremichael M., Forootan E., Wakbulcho G., Anyah R., Ferreira V.G., Alemayehu T. (2014). Characterization of Ethiopian mega hydrogeological regimes using GRACE, TRMM and GLDAS datasets. Adv. Water Resour..

[55] Chen N., Li R., Zhang X., Yang C., Wang X., Zeng L., Tang S., Wang W., Li D., Niyogi D. (2020). Drought propagation in Northern China Plain: A comparative analysis of GLDAS and MERRA-2 datasets. J. Hydrol..

[56] Friedl M., Sulla-Menashe D. (2022). MODIS/Terra+Aqua Land Cover Type Yearly L3 Global 500m SIN Grid V061.

[57] Liu Y., Wang J., Wang C., Liang S. (2020). Evaluation of the spatial extent and intensity of the vegetation growth in China. Remote Sens. Environ..

[58] Clark M.P., Fan Y., Lawrence D.M., Adam J.C., Bolster D., Gochis D.J., Hooper R.P., Kumar M., Leung L.R., Rasmussen R.M. (2015). Improving the representation of hydrologic processes in Earth System Models. Water Resour. Res..

[59] Xiong Z., Zhang Z., Gui H., Zhu P., Sun Y., Zhou X., Xin Q. (2024). Predicting time series of vegetation leaf area index across North America based on climate variables for land surface modeling using attention-enhanced LSTM. Int. J. Digit. Earth.

[60] Srivastava N., Hinton G., Krizhevsky A., Sutskever I., Salakhutdinov R. (2014). Dropout: A simple way to prevent neural networks from overfitting. J. Mach. Learn. Res..

[61] Zhang Q., Han Y., Li V.O.K., Lam J.C.K. (2022). Deep-AIR: A hybrid CNN-LSTM framework for fine-grained air pollution estimation and forecast in metropolitan cities. IEEE Access.

[62] Read J.S., Jia X., Willard J., Appling A.P., Zwart J.A., Oliver S.K., Karpatne A., Hansen G.J., Hanson P.C., Watkins W.D. (2019). Process-guided deep learning predictions of lake temperature. Water Resour. Res..

[63] Kingma D.P., Ba J. Adam: A method for stochastic optimization. Proceedings of the 3rd International Conference on Learning Representations, ICLR 2015—Conference Track Proceedings.

[64] Prechelt L., Orr G.B., Müller K.-R. (1998). Early stopping—But when?. Neural Networks: Tricks of the Trade.

[65] Kvålseth T.O. (1985). Cautionary note about R^2^. Am. Stat..

[66] Chai T., Draxler R.R. (2014). Root mean square error (RMSE) or mean absolute error (MAE)?—Arguments against avoiding RMSE in the literature. Geosci. Model Dev..

[67] Legates D.R., McCabe G.J. (1999). Evaluating the use of “goodness-of-fit” measures in hydrologic and hydroclimatic model validation. Water Resour. Res..

[68] Willmott C.J., Matsuura K. (2005). Advantages of the mean absolute error (MAE) over the root mean square error (RMSE) in assessing average model performance. Clim. Res..

[69] Sen P.K. (1968). Estimates of the regression coefficient based on Kendall’s tau. J. Am. Stat. Assoc..

[70] Mann H.B. (1945). Nonparametric tests against trend. Econometrica.

[71] Wenzel S., Cox P.M., Eyring V., Friedlingstein P.J. (2016). Projected land photosynthesis constrained by CO_2_ and temperature sensitivity. Nature.

[72] Piao S., Zhang X., Chen A., Liu Q., Lian X., Wang X., Peng S., Wu X. (2010). The impacts of climate change on water resources and agriculture in China. Nature.

[73] Vicente-Serrano S.M., Beguería S., López-Moreno J.I. (2010). A multiscalar drought index sensitive to global warming: The standardized precipitation evapotranspiration index (SPEI). J. Clim..

[74] Ogle K., Reynolds J.F. (2004). Plant responses to precipitation in desert ecosystems: Integrating functional types, pulses, thresholds, and delays. Oecologia.

[75] Sala O.E., Gherardi L.A., Reichmann L., Jobbágy E.G., Peters D.P. (2012). Legacies of precipitation fluctuations on primary production: Theory and data synthesis. Philos. Trans. R. Soc. B Biol. Sci..

[76] Gherardi L.A., Sala O.E. (2019). Effect of interannual precipitation variability on dryland productivity: A global synthesis. Glob. Change Biol..

[77] Piao S., Wang X., Park T., Chen C., Lian X., He Y., Bjerke J.W., Chen A., Ciais P., Tømmervik H. (2020). Characteristics, drivers and feedbacks of global greening. Nat. Rev. Earth Environ..

[78] Charru M., Seynave I., Hervé J.C., Collet C., Wigneron J.P. (2017). Recent growth changes in Western European forests are driven by climate warming and structured across tree species climatic habitats. Ann. For. Sci..

[79] Yan Z., Jing Y., Xiaole P., Liu S. (2025). Temporal dynamic of the net ecosystem exchange of carbon dioxide and responses to meteorological drivers in mountain forest ecosystem of southeastern China. Aerosol Sci. Eng..

[80] Bloom A.A., Exbrayat J.F., van der Velde I.R., Feng L., Williams M. (2016). The decadal state of the terrestrial carbon cycle: Global retrievals of terrestrial carbon allocation. Nat. Commun..

[81] Smith W.K., Reed S.C., Cleveland C.C., Ballantyne A.P., Anderegg W.R.L., Wieder W.R., Liu Y.Y., Running S.W. (2016). Large divergence of satellite and Earth system model estimates of global terrestrial CO_2_ fertilization. Nat. Clim. Chang..

